# Expression of SRY-related HMG Box Transcription Factors (Sox) 2 and 9 in Craniopharyngioma Subtypes and Surrounding Brain Tissue

**DOI:** 10.1038/s41598-017-15977-3

**Published:** 2017-11-20

**Authors:** Vivian Thimsen, Nora John, Michael Buchfelder, Jörg Flitsch, Rudolf Fahlbusch, Harald Stefanits, Engelbert Knosp, Marco Losa, Rolf Buslei, Annett Hölsken

**Affiliations:** 1 Department of Neuropathology, Friedrich-Alexander University Erlangen-Nürnberg (FAU), Erlangen, Germany; 2 Department of Neurosurgery, Friedrich-Alexander University Erlangen-Nürnberg (FAU), Erlangen, Germany; 3 Department of Neurosurgery, University Clinic Hamburg-Eppendorf, Hamburg, Germany; 4 Department of Neurosurgery, International Neuroscience Institute, Hannover, Germany; 5 Department of Neurosurgery, Medical University of Vienna, Vienna, Austria; 6 Ospedale San Raffaele, Department of Neurosurgery, Milano, Italy; 7 Department of Pathology, Sozialstiftung Bamberg, Bamberg, Germany

## Abstract

Stem cells have been discovered as key players in the genesis of different neoplasms including craniopharyngioma (CP), a rare tumour entity in the sellar region. Sox2 and Sox9 are well-known stem cell markers involved in pituitary development. In this study we analysed the expression of both transcription factors using immunohistochemistry in a large cohort of 64 adamantinomatous (aCP) and 9 papillary CP (pCP) and quantitative PCR in 26 aCP and 7 pCP. Whereas immunohistochemically Sox2+ cells were verifiable in only five aCP (7.8%) and in 39.1% of the respective surrounding cerebral tissue, pCP specimens appeared always negative. In contrast, Sox9 was detectable in all tumours with a significantly higher expression in aCP compared to pCP (protein, *p* < *0.0001;* mRNA p = 0.0484) This was also true for the respective tumour adjacent CNS where 63 aCP (98.4%) and six pCP (66.7%) showed Sox9+ cells. We further confirmed absence of Sox9 expression in nuclear β-catenin accumulating cells of aCP. Our results point to the conclusion that Sox2 and Sox9, seem to play essential roles not only in the specific formation of aCP, but also in processes involving the cerebral tumour environment, which needs to be illuminated in the future.

## Introduction

The sellar region represents an important interface of the human brain with close proximity to essential vascular and neural structures. It is at this precise and critical location that craniopharyngioma (CP), a group of rare intracranial epithelial tumours, occur. Although these neoplasms are designated as histologically benign (WHO-grade I) tumours^1^, their immediate proximity to crucial anatomical brain structures like the hypothalamus, the pituitary stalk, the cavernous sinus and the optic chiasm, makes them a major clinical and surgical challenge causing serious sequelae^2,3^. CP, which represent 2–5% of all primary intracranial neoplasms, can be divided in two subtypes, adamantinomatous (aCP) and papillary (pCP) CP, which differ in terms of histological, clinical, and genetic issues^4,5^. ACP, with their characteristic of nuclear β-catenin accumulating cell clusters^6–9^, represent the more frequent variant, occurring mainly during childhood and showing a higher rate of loco-regional recurrences^10^.

In contrast, pCP are almost exclusively found in adults and show a milder progression of disease^3^. Although the precise origin of CP still remains unknown up to this point, aCP are thought to derive from pituitary progenitor/stem cells representing embryonal remnants of Rathke’s pouch epithelium, whereas pCP are hypothesized to represent metaplasia of squamous epithelia^11,12^. The assumption that so-called cancer stem cells (CSC) are involved in CP-formation has also gained in importance in the last years^13^. CSC are thought to share many features of dormant stem cells. They have the ability to force self-renewal and differentiation, but are lacking balance in cell divisions a situation caused by the accumulation of genetic events^14–16^. One of the most well-known dysregulations are activating mutations in components of the Wnt-signalling pathway^17^. Mutations in *CTNNB1*, the gene encoding β-catenin, have been described in the vast majority of aCP samples^6,8^, and increased Wingless (Wnt) signalling in pituitary progenitor/stem cells in mice gives rise to pituitary tumours resembling aCP^12^. Furthermore, the expression of several markers of stemness, like OCT-4, KLF4, CD44 and CD133 (Prominin 1), has previously been described in aCP cell clusters showing nuclear β-catenin accumulations^18,19^.

Members of the Sry-related HMG (high mobility group) box (Sox) transcription factors are additional markers of stemness. 20 different factors, all divided into 9 subgroups (A–H, with B1 and B2)^20–22^ have been identified to date. Sox2 (SoxB1 group) is widely expressed in developing CNS and is essential for the development of the hypothalamic-pituitary axis, showing a uniform expression pattern in Rathke’s pouch^23–25^. Sox2 amplification has further been described in relation to different types of cancer^26–28^. Sox2+ stem/progenitor cells in the adult mouse pituitary further evinced tumour-inducing potential in a non-cell-autonomous manner^29,30^. Sox9 (SoxE group) plays various important roles in the development of cartilage^31–33^, sex organs^34^, and the CNS^35^. It has been described to be essential for the induction and maintenance of neural stem cells^36^ and also for the further promotion of the neuron-glia switch in the spinal cord^37^. To this date different studies about expression and role of Sox2 and Sox9 in CP development and maintenance exist but are based on only small numbers of tumour samples and information regarding the papillary subtype is rare^12,19,29,38,39^. However, data about the expression of Sox2 and Sox9 in the tumour surrounding brain tissue is lacking. In order to obtain detailed information about the expression of the stem cell factors in both human CP subtypes, we specifically investigated the immunohistochemical distribution pattern in a large cohort of tumour samples in association to the adjacent brain tissue and furthermore their gene expression using quantitative PCR. As several interactions of Sox2 and Sox9 with β-catenin, a member of the Wnt signalling pathway, were already described, we carefully examined the distribution pattern of these proteins^33,40,41^. In addition, we also investigated the co-expression of Sox2 and Sox9 with the oligodendrocyte transcription factor 2 (Olig2) as well as the glial fibrillary acid protein (GFAP) to define tumour surrounding cerebral microenvironment. Olig2 is known for determining oligodendrocyte differentiation, is expressed in glial precursor cells, and is also described to be involved in the formation of brain tumours, especially gliomas^42^.

## Results

### Very Low Expression of Sox2 in CP Subtypes Compared to the Tumour Surrounding Cerebral Tissue

**Figure 1:**
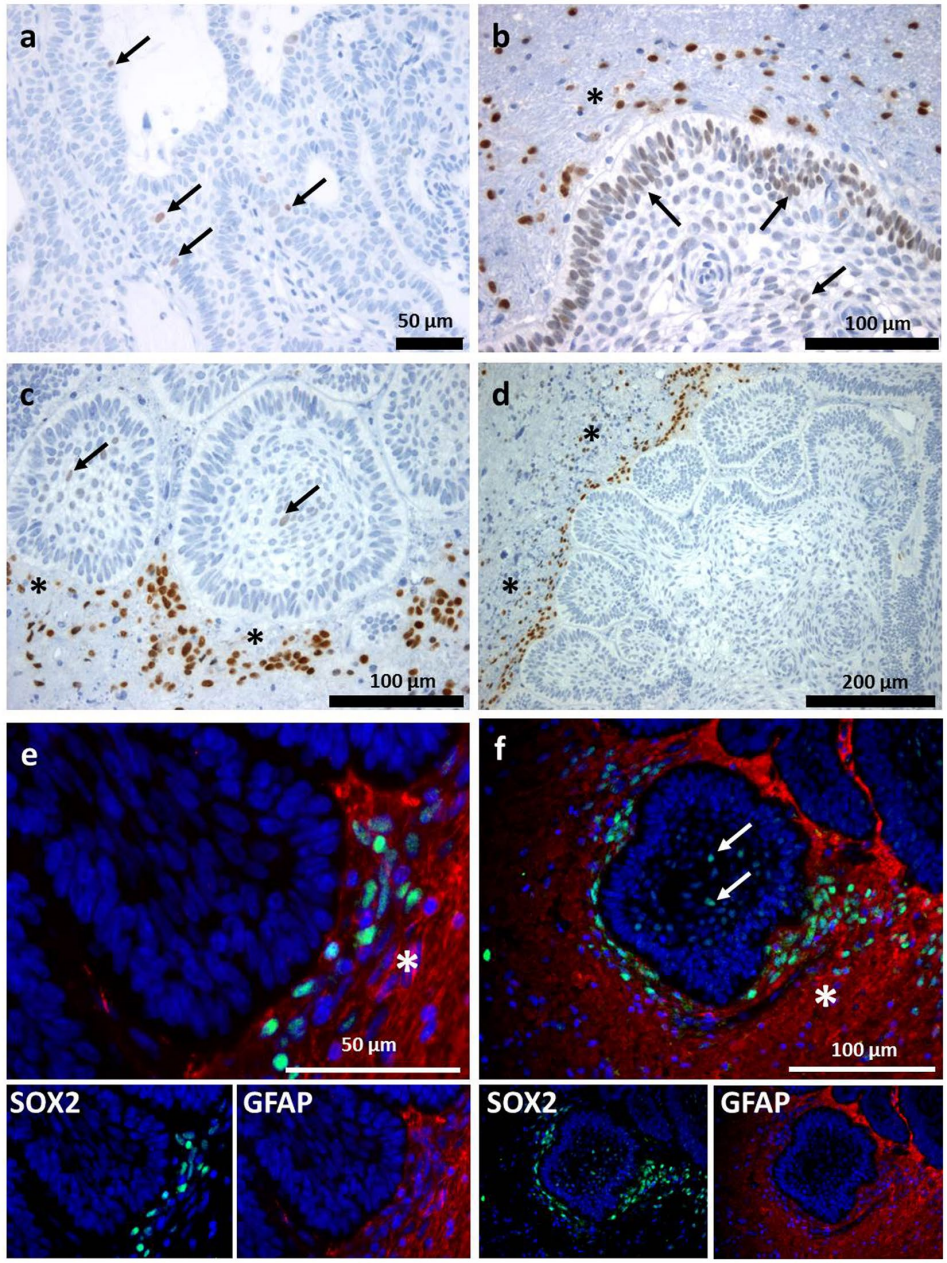
Immunohistochemical Expression Pattern of Sox2 in aCP and Surrounding Brain Tissue. (**a**–**c**) Nuclear Sox2 staining (arrow) was found within aCP tumour bulk and palisading cell layer and in cells of the tumour surrounding brain tissue (asterisk; **b**,**c**,**d**). (**e**,**f**) Merged double-immunofluorescence staining of Sox2 (green) and the glial marker GFAP (red) demarcating adjacent brain tissue (asterisk) of the tumour revealed Sox2+ cells being enhanced nearby the CNS-tumour junction. (f) The GFAP negative tumour tissue showed also Sox2+ cells (arrow). (ada27 = a, ada56 = b, ada48 = c,d,e; ada50 = f).

**Figure 2:**
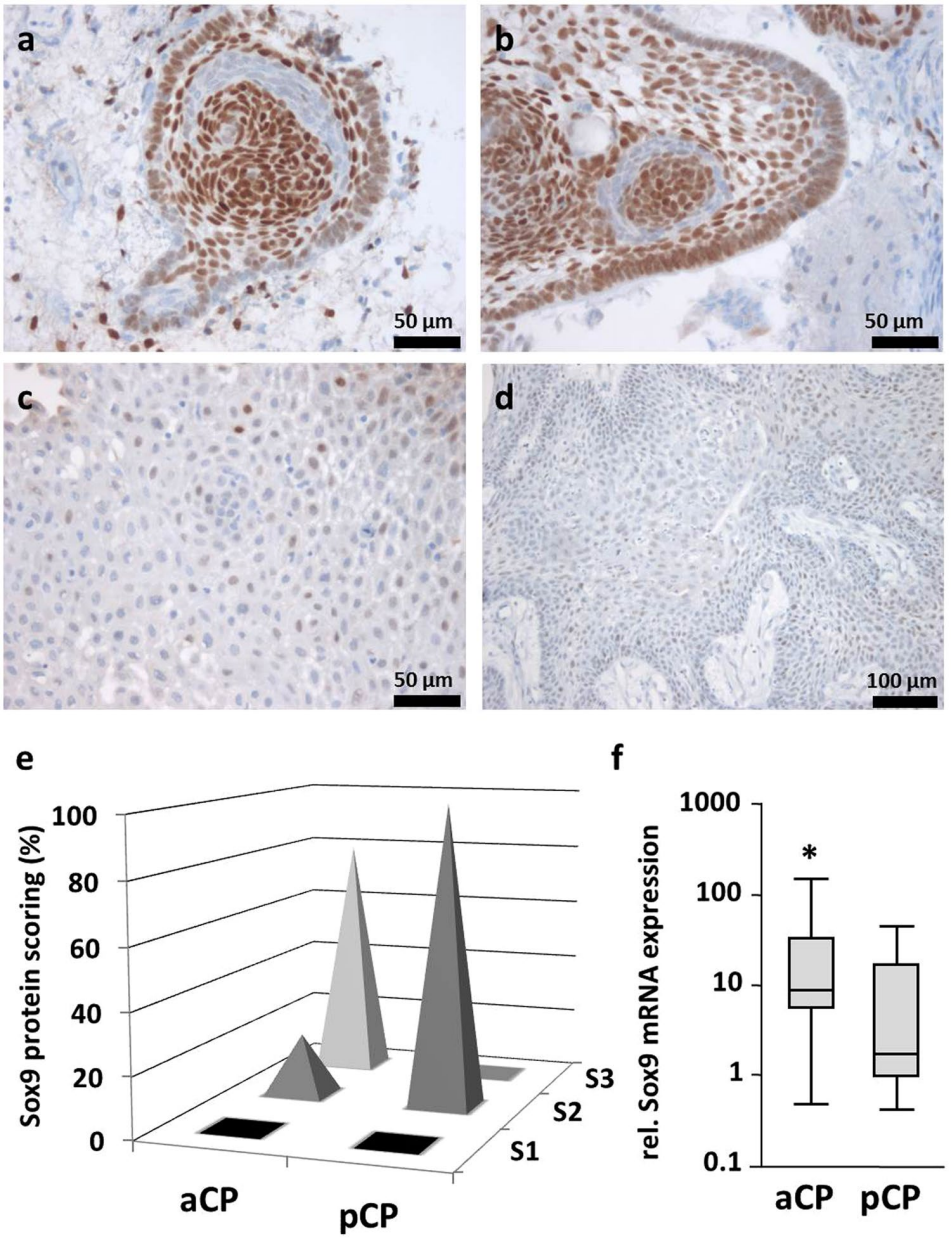
Immunohistochemical Staining of Sox9 in CP Subtypes and Summary of Calculated Sox9 Staining Scores. (**a**,**b**) Sox9 expression in aCP was not evenly distributed throughout the tumour, but rather showed significant regional differences (ada57). Although large proportions of the cells demonstrated a distinct nuclear staining pattern, small cell clusters in the direct vicinity were clearly negative. (**c**,**d**) Homogeneous and moderate immunohistochemical staining pattern of Sox9 in pCP (pap3, pap5). (**e**) The spikes illustrate the percentage shares of the immunohistochemical scoring groups (S1 = “no expression”; S2 = “low/moderate expression”; S3 = “strong expression”) within aCP and pCP specimens studied. Nuclear Sox9 staining was found in each of the 73 CP samples. Whereas all pCP samples showed only a moderate Sox9 expression (100% in group S2), aCP revealed expression in varying intensities (20.3% in group S2 and 79.7% in group S3) with a significantly higher overall Sox9 expression in this subtype [Fisher’s exact test (two-sided): *p < 0.0001*]. Exact values for each tumour sample were given in S-Table 1. (f) Sox9 mRNA expression analysis confirmed significantly enhanced levels in aCP (n = 26; mean = 24,47 in relation to pCP (n = 7; mean = 10,18; Mann-Whitney test, p = 0,0484).

We examined the distribution pattern of Sox2 in a cohort of 64 aCP and 9 pCP using immunohistochemistry. As part of our investigation, we considered the protein expression in both the tumour tissue as well as in the tumour adjacent brain parenchyma. When assessing the pCP specimens, we were unable to find any specific positive nuclear antigen reaction for Sox2 at all. In the group of aCP, a nuclear staining for Sox2 within the tumour tissue was hardly detectable as well. Only five cases (7.8%) revealed a few interspersed cells showing slight Sox2 expression (Fig. 1a,b,c). These cells were not only restricted to the cords, trabeculae and lobules of well-differentiated epithelium, but were also detectable in the palisading cell layer (Fig. 1b). However, the Sox2+ cells were obviously not localised within nodular whorls as was described for aCP reminiscent tumours in mouse models^29^. In contrast, we were able to prove a certain immunoreactivity in cells of the tumour surrounding brain tissue in 25 of the aCP samples (39.1%), as presented in Fig. 1(b,c,d). Double immunohistochemistry using the glial marker GFAP affirms that the occurrence of Sox2+ cells was restricted to occasional regions in the immediate vicinity of the tumour border where they often seemed to accumulate in certain areas of the cerebral microenvironment without showing a widespread distribution among the more distant brain parenchyma. Furthermore, it is remarkable that staining intensity of these Sox2+ cells was clearly higher than within the tumour tissue (Fig. 1b,c) The distribution pattern of Sox2+ cells is summarised and illustrated in Fig. 6a,b. Comparing the relative gene expression of Sox2 in aCP (n = 26; Median = 1.136) an pCP (n = 7; Median = 2.114) using quantitative PCR, the mRNA levels were almost equally distributed among aCP and pCP without any significant differences (S-Fig. 2a) [Mann-Whitney-test: α ≤ 0.05; *p = 0.3295*].

### Sox9 was Significantly Expressed in Both CP Subtypes and the Tumour Surrounding Brain Tissue

We also examined the distribution pattern of Sox9 in the same cohort of CP samples. As the tumours showed significantly higher amounts of protein expression overall, we defined a semi-quantitative total immunostaining score (TIS) for each case as described in detail in the methods section of this manuscript. Based on the calculated TIS, all specimens were subsequently divided into three different scoring groups (S1: TIS < 0; S2: TIS = 1–4; S3: TIS > 4). Although Sox9 staining was detectable in each tumour, there was, however, an obviously stronger antibody reaction perceptible in aCP compared to pCP. This observation was also reflected in the distribution of the scoring groups among both tumour subtypes, pointing to a significantly higher Sox9 expression in aCP [Fisher’s exact test (two-sided): *p < 0.0001*]. Whereas pCP showed only low to moderate Sox9 immunostaining levels (S2) in general, the majority (51 specimens; 79.7%) of aCP specimens revealed a strong staining pattern (S3). Examples demonstrating the varying strength of Sox9 immunoreactivity in both CP subtypes are shown in Fig. 2a–d. The respective scoring groups are illustrated in Fig. 2e. Even comparing the results from quantitative real time PCR, we could proof a significantly higher Sox9 mRNA expression rate in aCP (n = 26, Median = 8,98) compared to pCP (n = 7, Median = 1,728), (Fig. 2f) [Mann-Whitney-test (two-sided): α ≤ 0.05; *p = 0.0484*]. Comparing Sox2 and Sox9 expression in aCP, staining intensity implicate enhanced Sox9 levels which is also confirmed by mRNA expression levels (S-Fig. 2a). Sox9 mRNA expression levels of aCP in relation to Sox2 expression levels (n = 26, Median = 1.136) were significantly lower than Sox9 values (n = 26, Median = 8.98, Mann-Whitney-test (two-sided): *p < 0.0001*).

**Figure 3:**
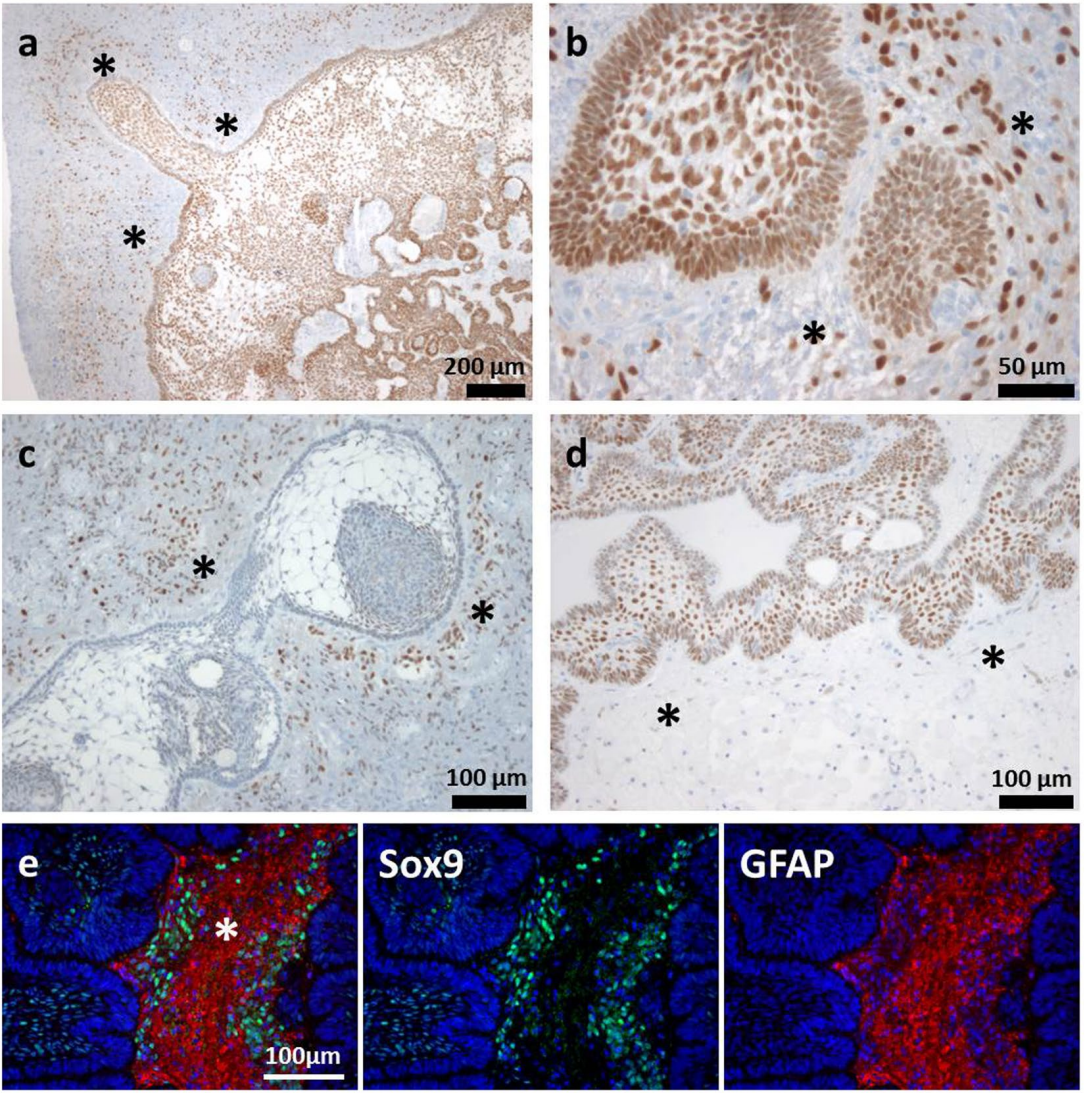
Varying Amounts of Sox9 Expression in aCP and Surrounding Brain Tissue. (**a**,**b**) Some cases showed a higher level of nuclear Sox9 expression in both tumour and surrounding brain tissue indicated by an asterisk [a = ada56 (TIS 12, BT 3), b = ada49 (TIS 12, BT 3)], whereas others (**c,d**) were predominantly stained in just one of the two. (**c**) ada28: Low Sox9 expression within the tumour tissue (TIS 3), distinct expression in the surrounding brain (BT 3). (**d**) ada27: High Sox9 expression scores within the tumour (TIS 12) and only occasional nuclear staining within the surrounding brain tissue (BT 1). (**e**) Merged double immunofluorescence staining of ada48 underscored enhanced levels of Sox9+ cells (green) within the tumour adjoining GFAP+ (red) brain tissue (asterisk). TIS = total immunostaining score, BT = staining score within tumour surrounding brain tissue.

In addition to Sox9+ tumour cells, we were also able to detect a strong nuclear Sox9 signal in several cells within the tumour surrounding GFAP+ cerebral tissue, both in aCP (98.4% of the patient samples) and pCP specimens (66.7% of the tumours). Once again, the average quantity and staining intensity was higher in the group of aCP samples when compared to pCP. The detailed scoring results for each tumour specimen are given in S-Table 1 and S-Fig. 1. It was evident that the amount and the intensity of positive cells in the tumour surrounding brain tissue did not correlate with the extent of Sox9 expression in the respective tumour tissue. Examples of the various combinations found in aCP are presented in Fig. 3a–d. Interestingly, we were able to find a significant association between high amounts of Sox9+ cells (>50%; BT3; n = 17) and the occurrence of Sox2+ cells (n = 12) in the aCP surrounding brain tissue [n(Sox2-) = 39; n(Sox2+) = 23; Fisher’s exact test (two-sided): *p = 0.0013*]. The CP subtype specific distribution pattern of Sox2 and Sox9 positive cells is summarised and illustrated in Fig. 6a–d.

**Figure 4:**
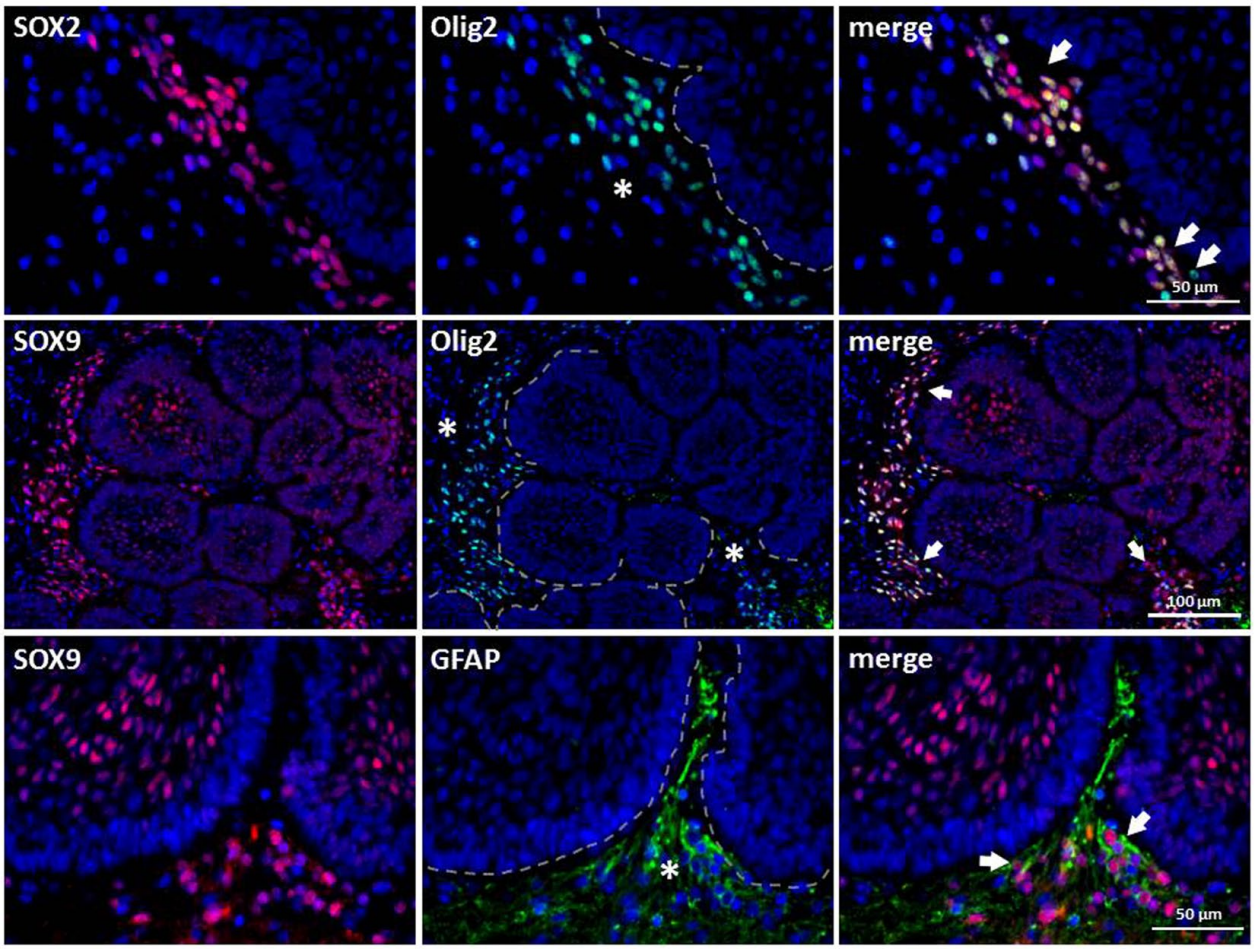
Co-expression of Sox2 and Sox9 with Olig2 in Cells of the Tumour Surrounding Brain Tissue. Double-immunofluorescence staining of ada48 showed that some but not all Sox2+ or Sox9+ cells (arrow) within the brain (asterisk) enclosing the tumour tissue (broken line) co-expressed Olig2. GFAP staining illustrates distinction between the epithelial tumour tissue (GFAP-) and adjacent brain tissue (GFAP+) with Sox9+ cells at the brain-tumour junction.

To gain more information about the properties of Sox2+ and Sox9+ cells located near the aCP, we decided to perform double-immunofluorescence staining of selected tumour samples using antibodies against Sox2 or Sox9 and Olig2. By doing so, we could prove a distinct co-expression of both proteins in several, but not all of the cells (Fig. 4). GFAP double immunohistochemistry was performed exemplary with Sox2 and Sox9 antibody to illustrate demarcation of the brain tissue (GFAP+) from tumour tissue (GFAP-) (Figs 1e,f; 3e; 4).

### Divergent Expression Patterns of Sox9 and β-catenin in aCP

**Figure 5:**
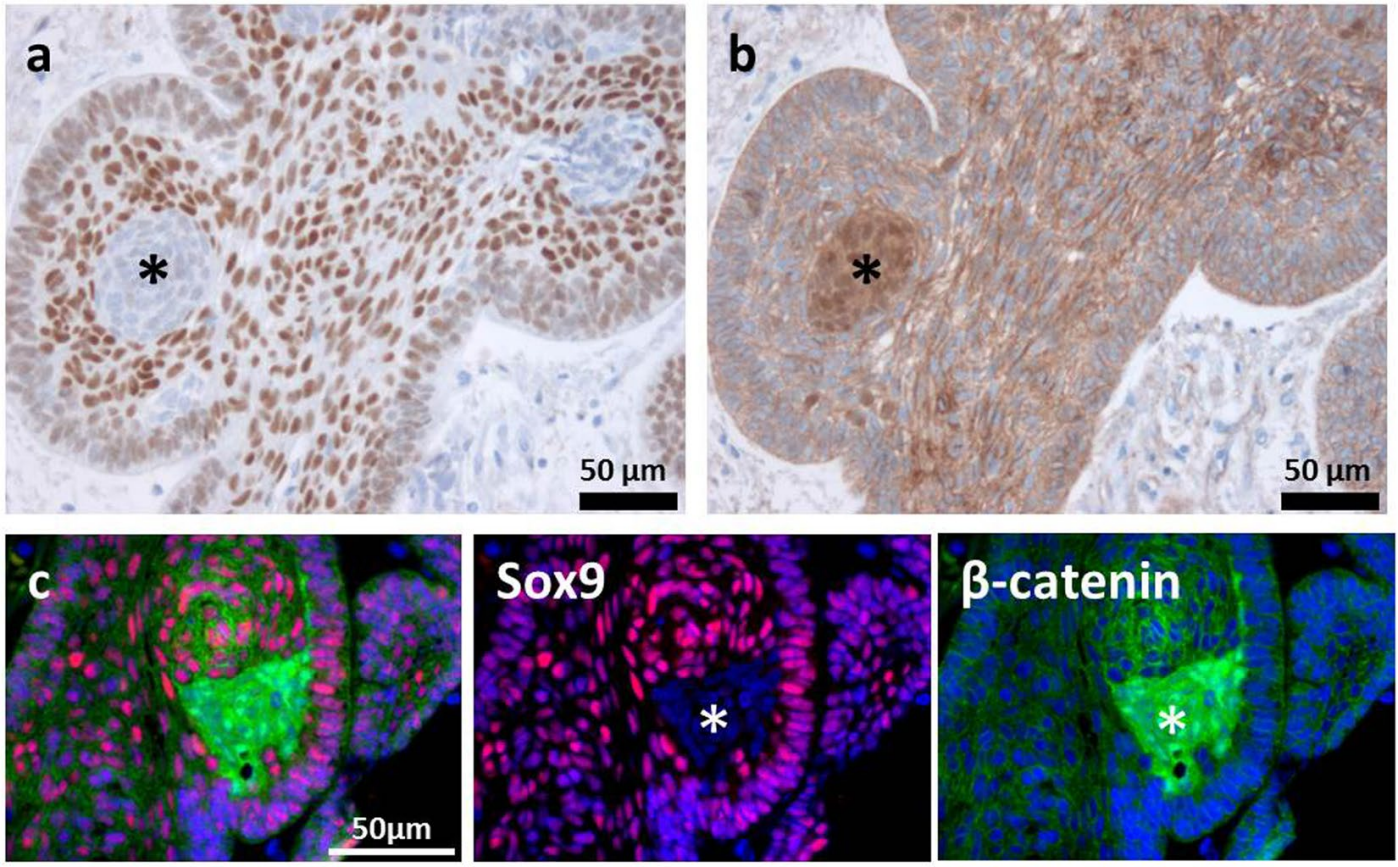
Correlation of Sox9 and β-catenin Expression in Paraffin-embedded Serial Sections and Double-immunofluorescence Staining of aCP. Immunohistochemical staining of serial sections using antibodies against Sox9 (**a**) and β-catenin (**b**) showed the absence of Sox9 in cell clusters with nuclear β-catenin accumulations (*); Examples taken from ada47. Merging double-immunofluorescence staining with antibodies against Sox9 and β-catenin (**c**), we were able to confirm the previous observation that cell clusters with nuclear β-catenin accumulations (green, asterisk) lack Sox9 expression (red, asterisk); Example was taken from ada50.

**Figure 6:**
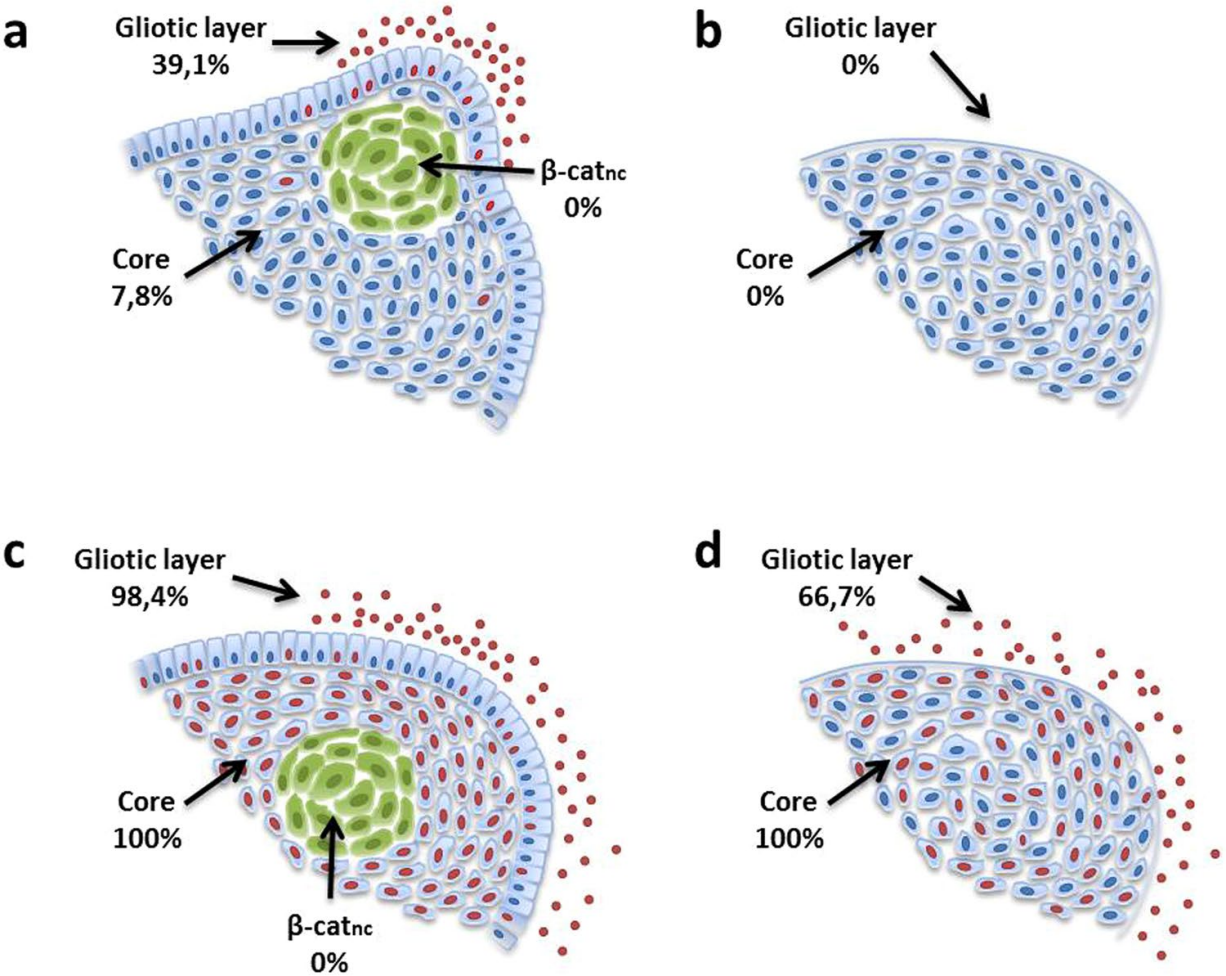
Percentage shares of Sox2 and Sox9 expression in aCP and pCP. Sox2 expressing cells could be detected in only 7.8% of aCP and in 39.1% of the respective surrounding gliotic layer (**a**). In the latter Sox2+ cells appeared especially surrounding finger-like tumour protrusions (**a**). pCP specimen appeared always negative (**b**). Sox9 could be found in all tumour samples with showing a significantly stronger staining pattern in the core of aCP (**c**) compared to pCP (**d**) (p < 0.0001). This was also true for the gliotic layer where 98.4% of aCP and 66.7% of pCP showed Sox9+ cells. Nuclear β-catenin accumulating cell clusters (green: β-cat_nc_), exclusively found in aCP always remained negative both for Sox2 and for Sox9.

During the analysis of the aCP samples, it became apparent that not all cells within the tumour bulk revealed nuclear staining for Sox9. Especially whirl-like cell clusters, typically showing nuclear β-catenin accumulations, completely lacked Sox9 expression, regardless of the case. In contrast, adjoining cells were characterised by a strong nuclear expression of the transcription factor, as documented in serial sections (Fig. 5a,b) and in double-immunofluorescence staining (Fig. 5c). Tumour cells of the palisading basal cell layer, bordering on adjacent brain tissue, revealed strong nuclear staining in most of the tumour samples as well (Fig. 5a,c). The distribution pattern of Sox9+ cells is summarised and illustrated in Fig. 6c,d.

### Association of Sox2 and Sox9 Expression and Clinicopathological Features

To address a potential prognostic value of Sox2 and Sox9 expression for patient care, we compared our results for gene expression (S-Fig. 2), and in case of Sox9 additionally the calculated staining scores, with several clinicopathological characteristics like age group, gender, and invasiveness. For none of those features a level of significance (p < 0,05) could be reached: Neither matching with relative gene expression results [gender: n(male) = 16; n(female) = 10; Mann-Whitney-test (two-sided): Sox2 *p = 0.8971;* Sox9 *p = 0.1089*, S-Fig. 2b *;* invasiveness: n(invasive) = 11; n(non-invasive) = 11; Mann-Whitney-test (two-sided): Sox2 *p = 0.5190*; Sox9 *p = 0.5190*, S-Fig. 2c; age group: n(adult) = 17; n(children) = 9; Mann-Whitney-test (two-sided): Sox2 *p = 0.4580*, Sox9 *p = 0.7916*, S-Fig. 2d], nor with Sox9 staining scores [age group: n(adult; age ≥19) = 41; n(children) = 23; Fisher’s exact test (two-sided): *p = 1.000*; gender: n(female) = 25; n(male) = 39; Fisher’s exact test (two-sided): *p = 0.1091*; invasiveness: n(invasive) = 19; n(non-invasive) = 23; Fisher’s exact test (two-sided): *p = 1.000*]. As the available clinical data concerning recurrence among our patient cohort was not extensive enough, we were unable to draw a reliable conclusion.

## Discussion

With rising insights in regulatory cell mechanisms, it has become clear that stem cells, pluripotency and developmental processes are not limited to the embryonic phase, but also that these play critical roles in tissue regeneration and tumour formation in mature tissues^43^. For this reason, the investigation of stem cell markers among neoplasms has increased over the course of the last few years. Sox2 and Sox9 are known as crucial transcription factors playing important roles in a great variety of different steps of embryo- and organogenesis and, in particular, in the development of the CNS and the hypothalamic-pituitary axis^36,44,45^. Both proteins have previously been described as being expressed in murine and human CP. As the presented results are based on just a small number of studies and tumour samples and provide no information about the expression of Sox2 and Sox9 in the tumour surrounding brain tissue^19,29,46^, we investigated the expression of Sox2 and Sox9 in a large cohort of aCP and pCP using immunohistochemistry (64 aCP, 9 pCP), double-immunofluorescence staining and quantitative PCR (26 aCP, 7 pCP). The immunohistochemical distribution pattern was analysed in detail in relation to β-catenin accumulating cell clusters and the tumour adjacent CNS tissue.

Sox2 represents an universal marker for multipotent neural stem cells both during embryogenesis and in adulthood^47^. The protein has been described as being involved in the pathogenesis of glial neoplasms^28^ as well as epithelial and mesenchymal tumours^26^. Its well-known role in the development of the hypothalamic-pituitary axis is reflected by a homogeneous expression in Rathke’s pouch epithelium^23–25^ and makes Sox2 a potential key player in the development of CP. This hypothesis was backed by the fact that targeted expression of oncogenic β-catenin in Sox2+ stem/progenitor cells in adult mouse pituitaries gives rise to tumours resembling human aCP^46^. In these mice, scattered Sox2+ cells were described within small β-catenin accumulating cell clusters representing a histomorphological hallmark of human aCP, but the expression was seen only temporarily^12,46^. Our data revealed, the rare Sox2+ cells found in five tumour samples were not located in or next to β-catenin accumulating cell clusters, confirming the results presented by Gaston-Massuet and colleagues^12^. They already suggested from the ACP mouse model that Sox2+ cells and the cell clusters showing nuclear β-catenin accumulations might be ontogenetically equal, but that these represent different stages of development^46^. Proving this thesis was not the focus of our investigation, but our results do not contradict this theory. Although, we were able to confirm identified Sox2 staining pattern in palisading cells and the tumour bulk, as described by *Garcia-Lavandeira et al*. our study dissent with observations describing the existence of individual Sox2+ cells in a high percentage (12/15; 80%) of CP specimens^19^. Therefore, we were hardly able to prove Sox2+ cells within the tumour tissue at all. Only few aCP cases (7.8%) and absolutely none of the pCP revealed antigen reaction. Interestingly, levels of Sox2 mRNA expression did not correlate with protein expression. As listed in S-Table 1 cases with Sox2 expression within the tumour exhibit mostly low mRNA levels, whereas others having high Sox2 mRNA levels were immunohistochemically negative (e.g. aCP6, aCP8). This discrepancy of mRNA and protein expression could be caused by several reasons like posttranscriptional mechanisms as earlier described^48^. A technical issue cannot be discarded since the Sox2 antibody used in our work is monoclonal rabbit anti-Sox2 antibody, while the Sox9 antibody is a polyclonal rabbit anti-Sox9 antibody. It is known that monoclonal antibodies are affected by protein posttranslational modifications.

However, the fact that we found Sox2+ cells in more than one-third of the tumours surrounding brain tissue may point to the recently suggested non-cell-autonomous role of Sox2+ stem/progenitor cells as observed in murine pituitary tumour formation^46^. These cells were predominantly located in the aforementioned tumour-specific cerebral microenvironment of aCP^49^ and often seemed to accumulate at certain tumour edges. We speculate that these areas are the biologically active parts of the lesion, inducing tumour outgrowth and causing subsequent hypoxia and injury to the surrounding brain tissue.

As Sox2 has not only been described to be involved in tumour formation itself but also in damage-induced reactions of the CNS, like reactive processes evoked by non-neuroepithelial CNS tumours^50^ and penetrating stab wounds^51,52^, we also examined the expression of Sox2 in relation to the CNS tissue markers GFAP and Olig2. GFAP is a main component of the cytoskeleton of glial cells, especially astrocytes and was used to define tumour surrounding brain tissue. Olig2 represents a transcription factor with important functions in the specification and differentiation of glial cells and neurons. Furthermore, Olig2 serves as a marker for progenitor cells and glioma stem cells (GSCs) and is required for glioma formation in a genetically relevant murine model^42,53^. Only recently, the existence of a new glial progenitor cell population was described, showing co-localization of Sox2 and Olig2 in the white matter of adult human brains. It was postulated that these cells may be responsible for tissue regeneration procedures^54^. In the GFAP positive tumour surrounding brain parenchyma of aCP, several Sox2+ cells showed co-expression of Olig2. Whether these Sox2+/Olig2+ cells represent de-differentiated astrocytes that acquire stem cell properties after injury^51^ or represent recruited progenitor cells from the perivascular niches and specific brain regions like the circumventricular organs^55^, remains unknown at this point. This important question and the function of Sox2+/Olig2+ cells in the formation and maintenance of aCP must be studied in depth in the future.

With regard to pCP, we could not detect any antigen reaction for Sox2 in cells of the tumour surrounding CNS tissue. One reason for this could be the different growth behaviour of pCP compared to aCP with a lower potential for brain infiltration, slower tumour growth, and, therefore, reduced damage of brain parenchyma with less reactive processes. The missing activation of Wnt- and Shh- signalling pathways in pCP, most likely involved in the aforementioned processes, could be a further explanation for this interesting observation.

The SoxE-group member Sox9 fulfils important functions during chondrogenesis, testis formation, neural stem cell migration and the development of pancreas and intestine. Its physiological functions aside, Sox9 has also been shown in conjunction with acquired diseases like fibrosis and cancer^40,56^. The specific role of Sox9 and its interaction with important signalling pathways have been best studied in chondrogenesis. Therefore, chondrocyte differentiation, proliferation, and maturation to hypertrophy are controlled by cross-talk between Sox9 and the canonical Wnt signalling pathway^33^. In non-physiological conditions, previous studies revealed the regulatory role of Sox9 on the transcription of Wnt genes in breast cancer cells^57^. Furthermore, it has previously been shown that overexpression of Sox9 is associated with the activation of Wnt/β-catenin signalling in brain tumours^40^. These facts are very interesting in relation to CP as β-catenin accumulating cells with subsequent Wnt signalling activation are the hallmark of aCP. In our study, it was precisely these cell-clusters which completely lacked Sox9 protein expression, while most of the surrounding tumour cells showed a strong antigen reaction. These results were consistent with those previously reported^12^.

This distribution pattern was significant in every single aCP specimen included in our study and confirms the results initially described in just a very small number of human tumour samples^12^. Using double-immunofluorescence staining and serial sections, this “punched-out” appearance in β-catenin accumulating cells became particularly obvious (Fig. 5) and indicates Sox9’s possible role in regulating the cellular distribution pattern of β-catenin in aCP. Independent of its DNA binding ability, nuclear localization of Sox9 is described to be both sufficient and necessary to enhance β-catenin phosphorylation and its subsequent degradation^41^.

Sox9 expression, in turn, has been described as being repressed by canonical Wnt/β-catenin, respectively^58–60^. In this process, SOXC genes (Sox4, Sox11, and Sox12) elevate the β-catenin level and repress Sox9 in the perichondrium and joints. Furthermore, SOXC proteins are able to stabilize β-catenin by replacing Sox9 and inhibiting GSK3 activity in the destruction complex^61^. It was already reported that Sox9 regulates the balance between self-renewal and differentiation during tumorigenesis. The protein is required for basal cell carcinoma (BCC) formation regardless of the oncogenic stimuli, the cell of origin, and the body location from where the tumour arises. Furthermore, it is responsible for the long-term maintenance of oncogene-expressing cells^62^ and Sonic hedgehog (SHH), fibroblast growth factor (FGF-), and transforming growth factor β (TGF-β)/bone morphogenic protein (BMP)-family members have shown an increase of Sox9 expression in different developmental processes^32,63^.

The fact that mitogenic signals of SHH, FGF, and BMP have been shown to be secreted from isolated nuclear β-catenin accumulating cells in mice and human aCP^46^ indicates that there might be further paracrine interactions between the clusters and the surrounding Sox9+ cells. These are very interesting observations that could give further insights into the formation and maintenance of aCP and should be examined in more detail on the molecular level in the future.

Sox9, analogous to Sox2, has been previously reported as participating in reactive glial processes of the CNS. In this regard, a strong signal for Sox9 could be proven in reactive gliosis in the cerebellum. In addition, SoxE-group members are further known to control different aspects of astrocyte and oligodendrocyte differentiation^63,64^. Considering the antigen reaction of Sox9 among the CP surrounding brain parenchyma, nearly all (98%) of the aCP and 67% of the pCP samples revealed a specific staining. In contrast to Sox2, the Sox9+ cells were not concentrated next to certain areas of the tumour, but rather widely distributed among all the adjoining brain parenchyma. The Sox9 antibody reaction was, however, stronger both in the aspect of quantity and as well as in the intensity in aCP compared to pCP. These differences, combined together with the fact that we were able to demonstrate Sox9+ cells, but no Sox2 antigen reaction in the brain tissue around pCP, could be declared compatible with the varying growth behaviour of both tumour subtypes and a consequently different extent of reactive gliosis in the tumour surroundings. Our findings lead to the hypothesis that Sox2+ and Sox9+ cells may represent different cell types that are involved at temporally varying points of CNS tissue regeneration. This interesting speculation must be the subject of future investigations.

We additionally could prove a significant correlation between high amounts of Sox9 and the appearance of Sox2 positive cell clusters within the tumour surrounding brain tissue. This may reflect the theory that the extent of tumour growth behaviour affects reactive CNS processes and vice versa. Although an overexpression of Sox9 has been associated with a poor clinical outcome of patients with malignant glioma^65^, comparing the protein and gene expression of both transcription factors with basic clinicopathological features (age groups, gender, and invasiveness) in CP patients showed no explicit association.

In summary, the stem cell transcription factors Sox2 and Sox9 seem to play essential roles not only in the formation of CP but also in processes involving the tumour surrounding brain parenchyma. Particularly interesting is the inverse relation of Sox9 expression and activation of the Wnt- signalling pathway in aCP. Immunohistochemistry clearly demonstrates a possible impact of Sox9 in the cellular distribution pattern of β-catenin being essential for subsequent Wnt target gene activation. In the future, these relations have to be investigated on molecular level in more detail.

## Methods

### Patient Cohort

In terms of immunohistochemistry, we analysed surgical specimens from 73 patients with CP, all of which were obtained as formalin fixed tissue from the archive of the Department of Neuropathology at the University Hospital Erlangen-Nürnberg. Appropriate surgeries have taken place in the Departments of Neurosurgery at the University Hospital Erlangen-Nürnberg, the International Neuroscience Institute in Hannover, the University Hospital Hamburg-Eppendorf, the Evangelic Hospital Bielefeld-Bethel, the University General Hospital in Thessaloniki (Greece), the General Hospital in Vienna (Austria) and the Hospital “San Raffaele” in Milan (Italy). The group of CP contained 9 pCP and 64 aCP, representing a correct representation of the higher frequency of this CP subtype. Each tumour sample was classified according to World Health Organization guidelines and only specimens containing sufficient amounts of the respective CP subtype were taken into account. The clinical data and detailed information concerning each tumour sample are provided in Supplementary Table 1 (S-Table 1).

### Ethical Approval and Informed Consent

All experimental protocols using human samples were approved by the Ethical Committee of the University of Erlangen-Nürnberg. Informed consent from all subjects was obtained. All used methods were carried out in accordance with the approved guidelines of the Ethical Committee of the University of Erlangen-Nürnberg and in accordance with the Declaration of Helsinki. A declaration of consent for further scientific investigation is available from each patient for all specimens as prescribed by the local ethics committee of the Friedrich-Alexander-University Erlangen-Nürnberg (FAU).

### Immunohistochemistry

3 to 4 μm thick sections of formalin-fixed and paraffin-embedded surgical samples were prepared, mounted on positively-charged object slides (Superfrost, Menzel, Braunschweig, Germany), and dried at 37 °C overnight. Immunohistochemical staining was performed using a staining machine (Benchmark ULTRA IHC/ISH Staining Module; Ventana Roche; Illkirch, France) and the streptavidin-biotin-staining system Ventana DAB following the manufacturer’s recommendations. Sox2 (SP76) was detected using a monoclonal rabbit-anti-Sox2 antibody (1:100; Cell Marque; Rocklin, CA, USA). A polyclonal rabbit-anti-Sox9 antibody (1:2500; Millipore; Temecula, CA, USA) was applied for visualization of Sox9. We utilised a monoclonal mouse-anti-β-catenin antibody (1:800; Clone 14; BD Biosciences; Franklin Lakes, NJ, USA) for detection of β-catenin. Olig2 expression was investigated using a monoclonal mouse-anti-Olig2-antibody (1:100; Clone 211F1.1; Millipore; Temecula, CA, USA). The staining procedures were verified by positive and negative controls (Sox2: squamous epithelium; Sox9: embryonic tissue; β-catenin: colon carcinoma; Olig2: normal brain, white matter).

### Double Immunofluorescence Staining

Double immunofluorescence stainings were carried out manually on selected tumour specimens using antibodies against Sox9 and β-catenin, Olig2 or GFAP (monoclonal mouse anti GFAP; 1:500; clone 6F2; Dako; Glostrup, Denmark) as well as Sox2 and Olig2 or GFAP. After dewaxing the slides, antigen retrieval by microwave pre-treatment was performed in citrate-buffer at pH 6. To prevent unspecific antibody binding, the slides then were incubated for two hours with a blocking solution containing PBS with foetal calf serum (5%; Biochrom AG; Berlin, Germany), goat serum (3%; Millipore; Temecula, CA, USA) and Triton ×100 (0,1%; Sigma-Aldrich; Steinheim, Germany). Primary antibodies were diluted in the blocking solution and incubated overnight at 4 °C. Carbocyanine 2 (Cy2) goat anti-mouse or goat anti-rabbit (1:100; green; Dianova; Hamburg, Germany) and Cy-3 goat anti-rabbit or goat anti-mouse secondary antibodies (1:100; red; Dianova; Hamburg, Germany) served as fluorescent markers, while cell nuclei were counterstained with Hoechst 33342 (Sigma Aldrich; Steinheim, Germany) at a concentration of 500 ng/ml for 5 min at room temperature. Slides were analysed using an Olympus BX-51 fluorescent microscope (Olympus; Hamburg, Germany) equipped with an F-View II CCD camera (Soft imaging systems; Stuttgart, Germany).

### Immunohistochemical Scoring

Sox2 and Sox9 antigen expression was evaluated by three independent observers (VT, AH and RB) using light microscopy (Olympus BX-50, Model U-MDOB, Olympus, Tokyo, Japan). In cases of discordant results, the specimens were re-evaluated on a double-headed microscope to attain consensus. Antigen expression was assessed as positive if specific nuclear staining of tumour cells could be found. Only vital tumour areas and intact surrounding brain tissue were taken into account for both quantity and intensity assessments, whereas areas with pronounced inflammatory and/or regressive tissue changes were omitted.

The expression of Sox9 was evaluated by calculating a total immunostaining score (TIS) following the assessment of quantity and intensity of the nuclear staining within the tumour as already described for other immunohistochemical markers^66^. A *TIS* is the product of a proportion score (PS), the estimated fraction of positive stained tumour cells (0 = 0%, 1 < 10%, 2 = 10–50%, 3 = 50–80%, 4 > 80%), and an intensity score (IS). The *IS* defines the estimated staining intensity (0 = no staining at all; 1 = weak staining; 2 = moderate staining; 3 = strong staining). The *TIS* ranges from 0 to 12 with only 9 possible values (0, 1, 2, 3, 4, 6, 8, 9, 12). The Sox9 total score was then divided into 3 scoring groups: scoring group 1 (S1, no expression, TIS = 0), scoring group 2 (S2, low/moderate expression, TIS = 1–4) and scoring group 3 (S3, strong expression, TIS > 4).

For Sox9, we additionally assessed the amount of nuclear antigen reaction in cells of the tumour surrounding brain tissue semi-quantitatively. To achieve an objective evaluation, we chose a similar score as previously described^49^, taking 4 different scoring groups into account (BT 0–4): BT 0, no immunopositivity; BT 1, individual Sox9+ cells (<10%); BT 2, moderate fraction of Sox9+ cells (10–50%); BT 3, high fraction of Sox9+ cells (>50%). S-Table 1 contains the respective scoring results of the tumour surrounding brain tissue for each sample.

With regard to Sox2, vital tumour areas and surrounding brain tissue were considered as well. As nuclear Sox2 staining within the tumour tissue could only be found in very rare cases, there was no need to create different quantity or intensity groups. This also applies for the environment, for which we only defined two distinct categories: “expression of Sox2” and “no expression of Sox2”.

### cDNA preparation

Total RNA of snap frozen tissue samples was isolated using TRIzol^®^ Reagent (Invitrogen, Life Technologies) according to manufacturers’ protocol or RNeasy^®^ extraction kit (Qiagen, Hilden, Germany). From all specimens, frozen sections were microscopically reviewed to confirm tumour content. After digestion with RNase-free DNase I (Invitrogen, Life Technologies) total amount of RNA was determined by measuring probes on a NanoDrop^®^ (Thermo Fisher Scientific, Waltham, Massachusetts), followed by reverse transcription, using SuperScript™ First-strand synthesis system (Invitrogen, Life Technologies) with oligo (dT) primers. Due to limitations regarding the availability of frozen tumour sampels or tumour size and tumour content collectives of immunohistochemistry and mRNA are not absolutely congruent.

### Quantitative Real Time PCR-Analysis

Relative quantification by qRT-PCR with Sybr Green II (Applied Biosystems, Life Technologies) was employed to assess the quantitative expression of Sox2 and Sox9 in whole-tumour tissue of 7 pCP and 26 aCP. To determine Sox expression relative quantification analyses were performed of CP cDNA. All analyses were carried out with the Applied Biosystems 7500 Fast Real-Time-PCR System (Applied Biosystems). Glyceraldehyde 3-phosphate dehydrogenase (GAPDH) was used as endogenous control of cDNA amount. Sequences of mRNA-specific primer employed in qRT-PCR analyses are listed in S-Table 2. To exclude nonspecific amplification, non-template controls for each primer were arranged on every plate and a melt curve analysis was performed. Analysis was conducted using the ΔΔC_T_-method according to manufacturers’ instructions. The case pCP3 was used as calibrator. All analyses were carried out in triplicates and evaluated statistically using Graph Pad software Prism 7.00 (La Jolla, California).

### Statistical Evaluation

In order to confirm the significance of differences in Sox9 protein expression between CP subgroups, Fisher’s exact test was conducted to ensure reliable results amongst the relatively small number of samples. The same test was also used to analyse certain associations between different clinical characteristics (e.g. age group, gender or invasiveness) of the patient cohort and a high Sox9 expression in aCP. Those analyses were conducted using IBM SPSS Statistics 23 (IBM, Armonk, NY, USA). The results of gene expression were analysed for normal distribution employing the Kolmogorov-Smirnov and the Shapiro-Wilks test using Graph Pad software Prism 7.00 (La Jolla, California). As Gaussian distribution could not be assumed, a Mann-Whitney test was performed for further statistical evaluation regarding potential associations with clinicopathological features e.g. age group, gender or invasiveness.

## Electronic supplementary material

Supplementary material
