# Supplementary material for: Expression of SRY-related HMG Box Transcription Factors (Sox) 2 and 9 in Craniopharyngioma Subtypes and Surrounding Brain Tissue
Source: Sci Rep. 2017 Nov 20;7:15856. doi: 10.1038/s41598-017-15977-3 (PMC5696533; doi:10.1038/s41598-017-15977-3)
Supplement: Supplementary file 1 — Supplementary material [file 41598_2017_15977_MOESM1_ESM.pdf]

Expression of SRY-related HMG Box Transcription Factors (Sox) 2 and 9 in Craniopharyngioma Subtypes and Surrounding Brain Tissue

Vivian Thimsen[1], Nora John[1], Michael Buchfelder[2], Jörg Flitsch[3], Rudolf Fahlbusch[4], Harald Stefanits[5], Engelbert Knosp[5], Marco Losa[6], Rolf Buslei[7*] and Annett Hölsken[1]

					Sox2			Sox9					
Patient	Sex	Age	Age Group	Invasive-ness	Tumor	BT	Rel. mRNA	PS (0-4)	IS (0-3)	TIS	Scoring Group (S1-S3)	BT (0-3)	Rel. mRNA
	pCP												
**pCP1**	m	52	a	no	-	-	3.1879	1	1	1	S2	0	3.0061
**pCP2**	m	19	a	no	-	-	0.9873	1	1	1	S2	2	1.6222
**pCP3**	f	49	a	no	-	-	1.0036	2	1	2	S2	2	1.0002
**pCP4**	m	57	a	-	-	-	7.0425	2	2	4	S2	2	1.7275
**pCP5**	f	46	a	no	-	-	0.7921	2	1	2	S2	1	0.4205
**pCP6**	m	40	a	yes	-	-	16.2004	2	1	2	S2	1	17.3091
**pCP7**	f	56	a	-	-	-	-	2	1	2	S2	2	-
**pCP8**	m	31	a	no	-	-	-	1	1	1	S2	0	-
**pCP9**	m	62	a	yes	-	-	2.1143	2	2	4	S2	0	46.1707
	aCP												
**aCP1**	m	48	a	no	-	-	-	3	3	9	S3	1	-
**aCP2**	m	9	p	-	-	-	-	2	3	6	S3	3	-
**aCP3**	m	50	a	no	-	-	1.0756	3	1	3	S2	1	28.9128
**aCP4**	f	3	p	-	-	-	-	3	2	6	S3	2	-
**aCP5**	f	29	a	yes	-	+	-	4	3	12	S3	2	-
**aCP6**	f	33	a	-	-	-	15.9290	2	3	6	S3	2	5.8463
**aCP7**	f	14	p	-	-	-	-	3	1	3	S2	1	-
**aCP8**	m	8	p	-	-	-	17.6287	3	3	9	S3	2	7.8862
**aCP9**	m	58	a	-	-	-	-	3	3	9	S3	2	-
**aCP10**	f	2	p	-	-	-	-	2	1	2	S2	2	-
**aCP11**	f	9	p	no	-	-	-	3	2	6	S3	1	-
**aCP12**	m	32	a	no	-	-	-	2	2	4	S2	1	-
**aCP13**	m	28	a	no	-	+	-	3	2	6	S3	2	-
**aCP14**	m	19	a	no	-	-	-	3	3	9	S3	2	-
**aCP15**	m	22	a	no	-	-	-	4	3	12	S3	3	-
**aCP16**	f	60	a	-	-	-	-	4	3	12	S3	2	-
**aCP17**	m	56	a	no	-	-	0.2786	3	2	6	S3	1	2.6231
**aCP18**	m	70	a	no	-	-	1.1968	4	3	12	S3	3	1.8866
**aCP19**	m	35	a	no	-	+	23.7424	3	3	9	S3	3	6.8355
**aCP20**	f	35	a	no	-	+	7.9801	2	2	4	S2	2	0.4827
**aCP21**	m	24	a	no	-	-	-	2	1	2	S2	1	-
**aCP22**	m	15	p	no	-	+	0.0993	3	2	6	S3	1	6.1443
**aCP23**	f	54	a	yes	-	-	0.2121	2	2	4	S2	1	37.0092
**aCP24**	m	49	a	-	-	-	-	3	3	9	S3	2	-
**aCP25**	m	13	p	no	-	+	-	3	3	9	S3	2	-
**aCP26**	f	4	p	yes	-	-	1.1695	3	3	9	S3	3	2.6062
**aCP27**	f	47	a	-	+	+	0.2955	4	3	12	S3	1	6.9234
**aCP28**	m	35	a	yes	-	+	-	3	1	3	S2	3	-
**aCP29**	f	4	p	yes	-	-	-	3	1	3	S2	2	-
**aCP30**	f	11	p	yes	+	-	0.5013	3	3	9	S3	1	7.0292
**aCP31**	m	39	a	no	-	-	-	3	2	6	S3	1	-
**aCP32**	m	7	p	yes	-	-	0.5865	3	2	6	S3	1	14.7976
**aCP33**	m	50	a	yes	-	+	5.6807	3	3	9	S3	2	12.3450
**aCP34**	f	6	p	-	-	-	-	1	1	1	S2	2	-
**aCP35**	m	37	a	yes	-	-	-	3	2	6	S3	2	-
**aCP36**	f	58	a	no	-	-	-	2	2	4	S2	1	-
**aCP37**	f	63	a	no	-	-	-	2	3	6	S3	2	-
**aCP38**	m	32	a	yes	-	-	4.1506	3	3	9	S3	1	21.5289
**aCP39**	m	8	p	yes	-	-	0.3211	2	2	4	S2	0	44.1824
**aCP40**	m	47	a	yes	-	-	1.6104	4	3	12	S3	1	155.0342
**aCP41**	m	17	p	yes	-	+	-	3	2	6	S3	3	-
**aCP42**	f	31	a	-	-	-	-	2	1	2	S2	2	-
**aCP43**	f	47	a	yes	-	+	0.8097	3	2	6	S3	2	5.5244
**aCP44**	m	3	p	no	-	-	-	4	3	12	S3	1	-
**aCP45**	f	5	p	no	-	+	0.1632	3	3	9	S3	3	3.2313
**aCP46**	f	60	a	-	-	+	-	3	3	9	S3	3	-
**aCP47**	m	72	a	no	-	+	0.7583	3	3	9	S3	2	76.8999
**aCP48**	f	16	p	no	+	+	2.5441	3	3	9	S3	3	78.7769
**aCP49**	f	38	a	yes	-	+	10.4515	4	3	12	S3	3	9.3488
**aCP50**	m	39	a	yes	-	+	1.8435	3	2	6	S3	2	23.5158
**aCP51**	m	1	p	-	-	-	-	3	3	9	S3	2	-
**aCP52**	m	58	a	no	-	+	0.8689	3	3	9	S3	2	33.8493
**aCP53**	m	48	a	no	-	-	0.3118	3	3	9	S3	2	34.3797
**aCP54**	m	28	a	yes	-	-	-	3	3	9	S3	2	-
**aCP55**	f	7	p	-	-	+	-	3	2	6	S3	3	-

**aCP56**	m	19	a	-	+	+	-	4	3	12	S3	3	-
**aCP57**	m	9	p	-	-	-	6.3787	4	3	12	S3	3	8.6119
**aCP58**	f	54	a	-	-	+	-	4	3	12	S3	1	-
**aCP59**	m	6	p	-	+	+	-	3	3	9	S3	3	-
**aCP60**	m	67	a	-	-	+	-	3	2	6	S3	3	-
**aCP61**	m	29	a	-	-	+	-	3	3	9	S3	2	-
**aCP62**	f	58	a	-	-	-	-	3	2	6	S3	*	-
**aCP63**	m	66	a	yes	-	+	-	4	3	12	S3	3	-
**aCP64**	m	7	p	yes	-	-	-	3	3	9	S3	*	-

S-Tab.1: Detailed clinical data and results of immunohistochemical Sox2 and Sox9 stainings as well as mRNA expression. This overview includes patient gender, age, age group, tumor invasiveness, detailed immunohistochemical evaluation data (PS= proportion score; IS= intensity score; TIS= total immunostaining score; BT = brain tissue) and relative mRNA expression (pap3 served as calibrator and was set as 1) for all patients with aCP and pCP included in this study. (*) marks two cases of aCP for which an evaluation of the surrounding brain tissue was not possible as there were only very small areas of normal brain parenchyma in the histological section.

Gene Symbol	Forward primer 5' - 3'	Reverse Primer 3' - 5'	Fragment length (bp)	Species
**GAPDH**	caacgaccactttgtcaagc	tcttcaaggggtctacatgg	237	human
**SOX2**	ttacgcgcacatgaacggct	tgcgagtaggacatgctgta	209	human
**SOX9**	atgaacgccttcatggtgt	tctcgctctcgttcagaagt	126	human

S-Tab. 2: Sequences of mRNA-specific primer employed in quantitative real time PCR analyses.

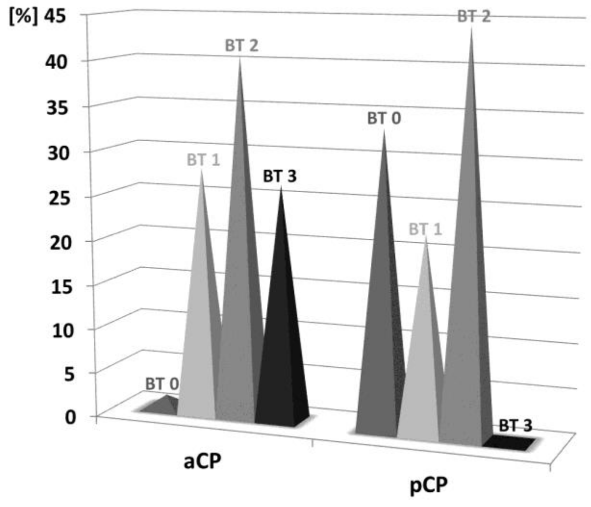

S-Fig.1: Percentage shares of the scoring results of Sox9+ cells in the tumor surrounding brain tissue. Sox9+ cells could be detected in the tumor-surrounding brain tissue of both CP subtypes with a stronger representation in aCP specimens. Exact aCP scoring data: BT 0 1.6%; BT 1 28.1%; BT 2 40.6%; BT 3 26.6%; 3.1% not available. Exact pCP scoring data: BT 0 33%; BT 1 22%; BT 2 45%; BT 3 0%. BT 0 = no immunopositivity; BT 1 = individual Sox9+ cells (<10%); BT 2 = moderate fraction of Sox9+ cells (10-50%); BT 3 = high fraction of Sox9+ cells (> 50%).

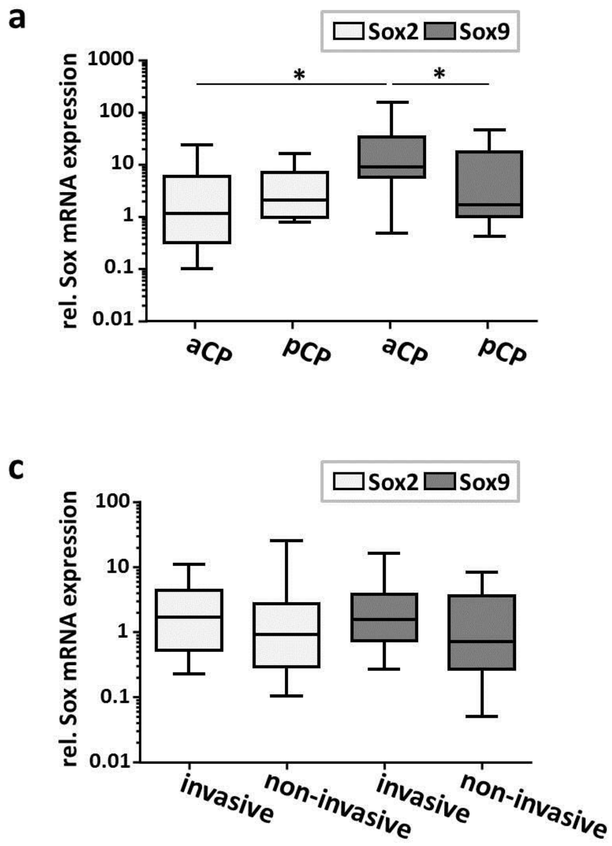

S-Fig. 2: Relative SOX2 and SOX9 gene expression in CP subtypes and analysis regarding clinical parameters in aCP .

(a) In contrast to SOX2 (p>0.05), SOX9 gene expression were significantly increased in aCP (n=26) in relation to pCP (n=7) (p=0.0484). Comparing gene expression of both transcription factors in aCP, SOX9 expression was also identified to be significantly increased in relation to SOX2 (p<0.0001). However, neither SOX2 nor SOX9 gene expression showed significant differences in aCP (p > 0.05) in matters of gender (b) (female: n=10, male: n=16), invasiveness (c) (invasive: n=11, non-invasive: n=11) and age group (d) (pediatric: n=9, adult: n=17). All statistical analyses were performed using the two sided Mann-Whitney test. Error bars are used to indicate the distribution range of single values.
